# Discrepancies Between Sex Prediction and Fetal Sex After Prenatal Noninvasive Cell-Free DNA Screening

**DOI:** 10.1210/jendso/bvaf007

**Published:** 2025-01-13

**Authors:** Selma F Witchel, Aleksandar Rajkovic, Svetlana A Yatsenko

**Affiliations:** Division of Pediatric Endocrinology, Department of Pediatrics, UPMC Children's Hospital, University of Pittsburgh, Pittsburgh, PA 15224, USA; Department of Pathology, University of California San Francisco, San Francisco, CA 94143, USA; Department of Obstetrics, Gynecology and Reproductive Sciences, University of California San Francisco, San Francisco, CA 94143, USA; Institute of Human Genetics, University of California San Francisco, San Francisco, CA 94143, USA; Department of Pathology, Stanford University, Stanford, CA 94304, USA

**Keywords:** cfDNA, differences in sex development, sex prediction, discrepant sex chromosomes, noninvasive prenatal screening, NIPS

## Abstract

In the last 10 years the field of prenatal diagnosis has been significantly reshaped followed by the implementation of noninvasive prenatal cell-free DNA (cfDNA) testing methodologies in clinical practice. Based on a superior performance and higher sensitivity and specificity than the former practice of biochemical markers screening, the American College of Obstetricians and Gynecologists and American College of Medical Genetics and Genomics recommend noninvasive prenatal cfDNA screening for trisomy 21, 18, 13, and sex chromosome aneuploidy to all pregnant people. While cfDNA screening is helpful in risk assessment for the most common autosomal trisomies, cfDNA also provides information about fetal sex chromosomes. Prediction of fetal sex is highly desired by the parents and also useful to healthcare providers for management of pregnancies that are at-risk for X-linked conditions. In fact, utilization of cfDNA screening has resulted in a significant number of referrals to evaluate discordant results for cfDNA sex prediction and appearance of fetal genitalia by prenatal ultrasound scan or at birth raising concerns about the fetus/infant atypical sex development known as a difference in sex development (DSD). In this mini-review, we outline principles and limitations of cfDNA technology, summarize recent findings related to cfDNA test performance in prediction of sex chromosome abnormalities and DSD conditions, define the technical and biological causes of discrepant results, provide recommendations to consolidate efforts by prenatal and clinical management teams in challenging situations, and discuss ethical considerations associated with fetal sex prediction and prenatal DSD diagnosis.

Prenatal diagnosis is crucial for monitoring fetal health and identifying potential genetic disorders. Traditionally, invasive procedures such as chorionic villus sampling (CVS) and amniocentesis have been used to diagnose genetic conditions at 10 to 13 weeks and after 15 weeks of gestation, respectively. However, these techniques carry a procedure-related risk of fetal loss in up to 1% of cases, causing concern for prospective parents and healthcare providers [1]. To minimize the need for invasive procedures and reduce the risk of pregnancy loss, noninvasive prenatal testing (NIPT), also known as cell-free DNA (cfDNA) screening, has emerged as an alternative to traditional prenatal screening methods such as maternal serum marker analysis for fetal trisomies 21, 18, and 13. Introduced into clinical practice in 2011, cfDNA screening analyzes cell-free placental DNA present in the bloodstream of a person during pregnancy [2]. This noninvasive technique has demonstrated high accuracy, specificity, and improved detection rates for common fetal trisomies. Due to its superior performance, the American College of Genetics and Genomics recommends prenatal noninvasive cfDNA screening for fetal trisomies 13, 18, 21, and X and Y sex chromosome aneuploidies to all pregnant individuals, regardless of age, with singleton and twin gestations [3-6].

In addition to facilitating the diagnosis of fetal chromosome disorders during gestation, prenatal cfDNA screening provides information about fetal sex, enabling better clinical management and planning by prospective parents and healthcare providers. However, this technology has limitations, particularly in predicting sex chromosome aneuploidies. Discrepancy between the fetal sex chromosome complement determined by cfDNA screening and the appearance of genitalia visualized by prenatal ultrasound or at birth has been reported in at least 1 out of 1500 to 2000 pregnancies [7-9].

These discrepancies can lead to stress and anxiety among expectant parents and their providers, often requiring consultation and additional workup by endocrinology physicians to rule out a difference in sex development (DSD) condition. The conventional notion that sex can only be binary leads to angst and trepidation. Such situations benefit from prompt consultation by pediatric endocrinologists, pediatric urologists, and geneticists to assess for a DSD.

The DSDs comprise a group of congenital conditions characterized by atypical development of chromosomal, gonadal, or anatomic sex, with an estimated incidence of 1 in 4500 to 5500 births [10]. Some DSDs are evident at birth due to atypical external genitalia, while others may be diagnosed during adolescence or adulthood. DSDs are classified into 3 broad categories: sex chromosome aneuploidy, 46,XY DSD, and 46,XX DSD. Turner syndrome and Klinefelter syndrome are examples of sex chromosome aneuploidy. Individuals with androgen insensitivity or defects in testosterone biosynthesis are included in the 46,XY DSD category. The 46,XX DSD category included virilized females with congenital adrenal hyperplasia. Thus, the sex chromosome karyotype, hormone values, and anatomic sex may be incongruent.

During the initial stages of sex development, the gonads, internal genital ducts, and external genital structures develop from bipotential embryologic tissues, with the presence or absence of the Y chromosome directing the developmental trajectory of the undifferentiated gonad to become a testis or an ovary, respectively.

This mini-review focuses on the technical and biological causes of discrepant fetal sex determination and sex chromosome findings by prenatal noninvasive cfDNA screening. It provides examples of fetal and inherited chromosomal conditions that can result in false-positive (FP) and false-negative (FN) interpretations of cfDNA screening, aiming to assist healthcare professionals in understanding and managing these challenging situations.

## Literature Search Strategy

A literature search in the PubMed and Google Scholar databases was performed up to May 2024, to identify large cohort studies that assessed the clinical performance and rates of FP and FN noninvasive prenatal screening (NIPS) results for sex chromosomes. We also searched for individual cases and case series articles, reporting prenatal cfDNA findings in patients with DSDs. The following keywords and combinations of them were used to retrieve articles of interest: noninvasive prenatal screening, NIPT, NIPS, cell free DNA, cfDNA, false positive noninvasive screening, false negative noninvasive screening, prenatal diagnosis of DSD, discordant sex chromosomes, sex discordance, sex chromosome aneuploidy, unbalanced X;Y translocation, X/Y translocation, external genitalia, ambiguous genitalia, gonadal dysgenesis, *SRY-*positive female, differences of sex development, DSD, sex reversal. Articles published in the English language were reviewed to ascertain incidence and causes of discordant prenatal cfDNA sex chromosome results with the appearance of the external genitalia by fetal ultrasound or at birth.

## Prenatal Noninvasive cfDNA Screening

**Figure 1.**
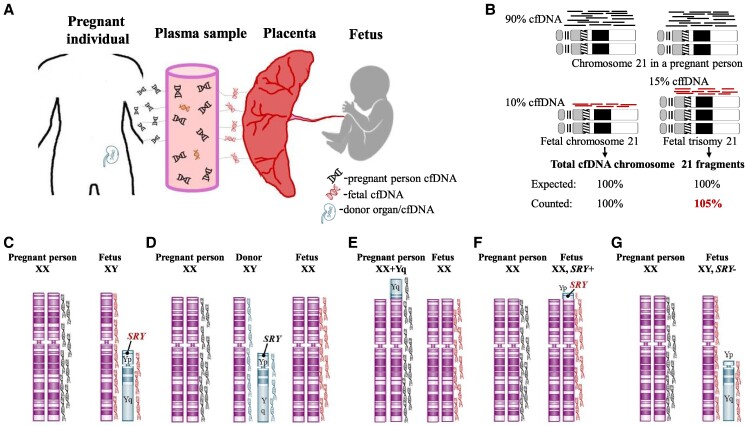
Principles of prenatal noninvasive cfDNA screening. (A) During pregnancy, the bloodstream contains a mixture of cfDNA fragments derived from cells present in the body of a pregnant individual, which may include cells from normal tissue, neoplasia, donor tissue, and placenta. (B) NIPS using the counting-based approach. This algorithm operates on the principle that the whole human genome is equally represented in the plasma of a pregnant person and cfDNA is proportionate to the genomic size and number of chromosomes. In a 10-week diploid pregnancy, cfDNA from a fetal chromosome 21 is expected to represent ∼10% of the total chromosome 21 cfDNA fragments. If the fetus has trisomy 21, more cfDNA sequences from chromosome 21 will be present in plasma, resulting in an increased percentage of the total chromosome 21 cfDNA fragments compared to a normal fetus or the cfDNA percentage for another chromosome. (C) Sex chromosome complements in the pregnant individual (XX) and fetus (XY) contributing to the cfDNA pool in plasma. Black cfDNA molecules are derived from the body of the pregnant person, while red cfDNA fragments are fetal in origin. (D) In cases of an organ transplant, donor-derived Y chromosome cfDNA fragments (in blue) are incorrectly interpreted as fetal, leading to a false-positive fetal sex prediction. (E) False-positive fetal sex prediction and sex chromosome aneuploidy (XXY) caused by detection of Y chromosome cfDNA fragments present in the cells of the pregnant individual with 2 copies of the X chromosome in the fetus. (F) Fetus with an X–Y chromosome rearrangement resulting in 46,XX,*SRY*+ male syndrome. Depending on the cfDNA screening methodology used, fetal sex might be predicted as female (XX) when the Y-derived segment is too small to detect, or male with a risk for XXY sex chromosome aneuploidy. (G) True-positive cfDNA findings in an XY fetus with discordant genital appearance, affected by a DSD (loss of the *SRY* gene).

Over the last 2 decades cfDNA analysis has transformed prenatal genetic testing by enabling the screening for fetal chromosome aneuploidies without posing a direct harm to the fetus in all pregnant patients regardless of age. cfDNA molecules are short DNA fragments primarily derived from the apoptosis of hematopoietic cells, but they can also originate from other tissues, including adipose tissue and various other cell sources. In pregnant people, cfDNA also originates from fragmented placental trophoblastic cell [4]. Since the placental trophoblast and fetus develop from the same embryo, their genetic makeup is usually identical. Therefore, placental cfDNA is commonly referred to as fetal cfDNA (cffDNA). Circulating cfDNA in a pregnant person is a mixture predominantly composed of cfDNA from the person's own cells and cffDNA molecules (Fig. 1A). Although cffDNA can be detected in plasma as early as at 4 weeks of gestation, NIPS is commonly performed at 10 to 12 weeks of gestation when the proportion of cffDNA (fetal fraction [FF]) is expected to reach about 10% of a total cfDNA (Fig. 1A).

Currently, 2 main approaches are used for prenatal noninvasive cfDNA screening: massively parallel shotgun sequencing (MPSS) and single nucleotide polymorphism (SNP)–based methodology [11, 12]. The most common approach to NIPS involves utilizing MPSS and analyzing the data with a counting-based algorithm. This process includes the following major steps: (1) obtaining plasma from a blood sample of a pregnant person, (2) isolating cfDNA, (3) MPSS of cfDNA fragments, (4) assignment of each unique cfDNA sequence read to a specific chromosome, (5) counting a total number of cfDNA molecules per chromosome, (6) bioinformatic analysis and calculation of a Z-score that defines a degree of deviation from expected reference results (Fig. 1B). This approach is also used for determination of fetal sex chromosomes. The number of X chromosomes is calculated similarly to autosomes. In the presence of the Y chromosome cfDNA fragments, the fetus is likely a carrier of the Y chromosome (Fig. 1C). Subsequently, the fetal gender is interpreted as male, especially when the number of the Y chromosome cfDNA molecules corresponds to the estimated FF value. Based on cfDNA analysis for the X and Y chromosome, laboratories may report fetal XX (female) or XY (male) normal results or indicate that the screen is positive or at risk for sex chromosome abnormalities including monosomy X (Turner syndrome), XXY (Klinefelter syndrome), XXX (triple X syndrome), and XYY (Jacobs syndrome). The MPSS approach does not allow for the study of fetal cfDNA alone and, due to technical or biological reasons, may lead to FP and FN results.

NIPS is offered to many individuals who might have otherwise avoided fetal genetic testing. However, despite reports of high sensitivity and specificity, NIPS results can sometimes differ from the phenotype and postnatal karyotype. Many pediatricians are unfamiliar with the intricacies of this screening method and may struggle to interpret or further investigate potential discrepancies. These uncertain situations can generate anxiety for parents and apprehension for physicians, especially when the fetal external genitalia appear different from the NIPS karyotype results. With the increased use of NIPS, referrals to pediatric endocrinologists and geneticists have risen for evaluating infants with inconsistencies between NIPS results indicating sex chromosomal aneuploidies and the infant's apparent external genital appearance.

## False-Positive NIPS Results

There are several reasons why prenatal cfDNA screening may produce incorrect results. Regarding Y chromosome detection, FP NIPS results indicate the presence of the Y chromosome cfDNA in the plasma of a pregnant person, while the fetus actually lacks Y chromosome–specific DNA segments (Table 1). In such cases, NIPS findings may be reported as normal XY, high-risk XXY or XYY though the baby's karyotype is normal 46,XX or abnormal (45,X or 47,XXX, for example). Such situations may come to the providers’ attention if female appearing genitalia are seen by prenatal ultrasound or at birth, or if preimplantation genetic testing or a concurrent genetic testing yields different sex chromosome results. Initially, a sample mishandling should be excluded. A FP result might occur due to errors in the collection or labeling of the samples, sample mix-up during the processing, misinterpretation of test results, or incorrect reporting.

**Table 1.** Discrepant findings of female genitalia by ultrasound and cfDNA positive for the Y chromosome

Possible causes	Recommended actions
**False-positive cfDNA (fetus is negative for the Y chromosome)**	
1. Sample processing error	Contact the laboratory, repeat cfDNA, repeat US
2. Pregnant person as a source of the Y chromosome history of a bone marrow or organ transplant Yq rearrangements (negative for the *SRY* gene) XX/XY Chimerism or XX/XXY mosaicism	Verify patient's medical history, perform G-banding karyotype analysis on the pregnant person, FISH or PCR-based study for the Y chromosome mosaicism
3. Placental source of the Y chromosome Vanishing XY twin 46, XY/46, XX chimerism	Postnatal testing: FISH analysis for X and Y chromosomes on placental tissue
**True-positive cfDNA (the Y chromosome DNA is present in placenta and/or fetus)**	
1. The Y chromosome mosaicism (fetal/placental discordance such as 45, X/46, XY or 46, XX/47, XXY)	Prenatal diagnosis: amniocentesis, karyotype, FISH using the *SRY* probe, CMA Postnatal testing: Peripheral blood karyotype analysis, FISH using the *SRY* probe, FISH and CMA as clinically indicated, additional studies to rule out sex chromosome mosaicism and monogenic causes of DSDs
2. Fetal source of the Y chromosome DNA Yq gain due to unbalanced X-Y translocations Yq gain due to Yq-autosome translocations	
3. DSD—XY female *SRY* gene deletion, Y chromosome rearrangements Complete androgen insensitivity Other molecular causes of isolated DSD Syndromic DSD (female genitalia + birth defects)	

Abbreviations: cfDNA, cell free DNA; CMA, chromosomal microarray analysis; DSD, difference in sex development; FISH, fluorescence in situ hybridization; PCR, polymerase chain reaction; US, ultrasound.

Discordant results might be explained by determining the source of the Y chromosome in the plasma of a pregnant person. Rather than being fetal in origin, the Y chromosome or its segments may originate from the pregnant individual rather than the fetus. A common cause of FP results is a history of an organ transplant (bone marrow, kidney, liver, etc.) from a donor with an XY chromosome complement (Fig. 1D) [13]. Patients themselves might be unaware of a transplantation history if the procedure occurred in childhood. Similarly, a pregnant person might be affected by a sex-discordant constitutional chimerism [14], which occurs when an organism contains cells combined from 2 distinct zygotes. Chimerism in humans is rare and often observed among monochorionic dizygous twins as a result of twin to twin blood transfusion, but its incidence is likely underestimated. More frequently, chimerism is diagnosed in children with gonadal dysgenesis; however, healthy pregnant adults with 46,XX/46,XY chimerism have also been reported [15, 16].

Pregnant individuals may have Y chromosome material in all cells but that does not affect their ability to carry a pregnancy [17, 18]. Some patients have been diagnosed with unbalanced translocations between the X and Y chromosomes or a Yq/autosome rearrangement after the birth of an affected child or NIPS findings suggestive of a fetal sex chromosome aneuploidy. No evidence exists suggesting that the presence of Y chromosome segments lacking the *SRY* gene in cells with 2 X chromosomes has any effect on the development of ovaries and female reproductive organs [18]. Moreover, there are reports of a mutigenerational familial transmission of unbalanced X–Y translocations carrying *SRY* from fertile phenotypic females [18]. When an *SRY*-positive Y chromosome segment is attached to the X chromosome it might be completely silenced due to skewed X chromosome inactivation [19]. Pregnant people with unbalanced X–Y translocations, particularly involving the long arm of the Y chromosome (Fig. 1E), are more likely to receive FP NIPS results [18, 20, 21]. Although the incidence of chimerism, unbalanced X–Y translocations, and other instances when healthy pregnant individuals carry a portion or the entire Y chromosome has not been established and considered to be low in the general population, the cumulative frequency is greatly underestimated.

Another explanation for sex chromosome discordance between the placenta and the remaining fetus is the disappearance of one of the fetuses in multifetal dizygous gestations, known as vanishing twin syndrome. NIPS using SNP-based methodology that can distinguish cffDNA molecules from other cfDNA sources should be offered to transplant recipients, pregnant individuals using a donor egg or donor embryo, and those with multiple gestations [14].

## False-Negative NIPS Results

Contrary to FP NIPS, FN screening fails to detect the Y chromosome cfDNA in the plasma of a pregnant person, while in reality the fetus is the carrier of a segment or the entire Y chromosome (Table 2). As a result, NIPS findings might be misinterpreted as a normal XX or with a risk for monosomy X (45,X), but the fetal karyotype is normal 46,XY or abnormal. Besides sample processing errors, FN results occur due to technical limitations of the test.

**Table 2.** Discrepant findings of male or ambiguous genitalia by ultrasound and cfDNA negative for the Y chromosome

Possible causes	Recommended actions
**False-negative cfDNA (fetus is positive for the Y chromosome)**	
1. Sample processing error	Contact the laboratory, repeat cfDNA, repeat US
2. Low fetal fraction (<4%) in cfDNA testing	Repeat cfDNA
3. Placental/Fetal discordance Vanishing XX twin Y chromosome mosaicism (46, XX/47, XXY) 46, XX/46, XY chimerism	Collect prenatal history, perform FISH analysis for X and Y chromosomes on placental tissue if available
4. Chromosomal causes of *SRY*-positive XX male DSD Translocations between X and Y chromosomes such as der(X)t(X;Y)(p22.33; p11.31) Yp autosome translocations	Karyotype analysis, FISH analysis using the *SRY* FISH probe
**True-negative cfDNA (the Y chromosome DNA is not present in placenta and fetus)**	
1. Congenital adrenal hyperplasia	Targeted gene sequencing
2. *SRY*-negative isolated testicular/ovotesticular XX male DSD	
3. Syndromic conditions with urogenital anomalies (genital ambiguity + birth defects)	
4. Gestational hyperandrogenism Fetal exposure to exogenous androgens Maternal androgen-producing tumors	

Abbreviations: cfDNA, cell free DNA; DSD, difference of sex development; FISH, fluorescence in situ hybridization; US, ultrasound.

The use of cfDNA is complicated by various challenges. Typically, the amount of cffDNA positively correlates with gestational age. FF, the proportion of cffDNA to the total cfDNA fragments in the plasma, ranges from less than 4% to 30% [2, 22]. The FF is an important quality control and statistical factor affecting the accuracy of NIPS data interpretation. Laboratories use different methods for calculation of FF. A minimum of 4% FF is required to provide a reliable NIPS result; however, FN results in samples with up to 8% FF have been reported in literature [23]. Assessment and inclusion of FF in NIPS reports are recommended by professional guidelines. Multiple factors are known to negatively influence FF, including increased weight/body mass index of a pregnant person, medical condition, pregnancy complications, placental pathology, and use of medications (antibiotics, antiviral, antidiabetic, antithyroid, chemotherapy, aspirin, and others) [24].

Mosaicism confined to the placenta, accompanied by discordance between the placenta and the fetus, may also result in incorrect interpretation if the Y chromosome is absent among placental trophoblastic cells. In cases of a vanishing twin, chimerism, and sex chromosome mosaicism (46,XX/47,XXY; 45,X/46,XY; 45,X/47,XYY, and other karyotypes), 2 chromosomally different cell lines may be unequally distributed between trophectoderm and inner mass cells during the early stages of embryogenesis, leading to a placental/fetal discordance for sex chromosome complement. In the placenta, all cells might be positive for monosomy X, but the fetus itself could be mosaic or can contain a normal or a structurally abnormal Y chromosome [25].

A cryptic structural rearrangement involving the short (p) arm of the Y chromosome encompassing the *SRY* (sex-determining region, MIM*480000) gene is another cause for discrepant findings between NIPS and phenotype. The *SRY* gene is located on the terminal Yp, adjacent to the pseudoautosomal Xp/Yp region. Yp translocations to the X chromosome (Fig. 1F) or an autosome is a well-known cause of the *SRY*+ 46,XX testicular DSD (MIM#400045). In such conditions, there is no genetic discordance between the placenta and the fetus, both contain 2 X chromosomes and a small Yp segment, although due to the small size of the Yp-specific DNA segment the number of cffDNA molecules might be below the detection threshold by NIPS. Postnatal follow-up genetic testing of children born following a prenatal diagnosis of any sex chromosome aneuploidy is recommended to correctly define the child's karyotype, and rule out mosaicism and structural X or Y chromosome rearrangements [25, 26].

## True-Positive and True-Negative NIPS Results

For parents and healthcare providers, fetal sex determination is an important aspect of family planning. NIPS is also utilized for management of pregnancies with an increased risk for X-linked and sex-limited conditions. Prenatal noninvasive cfDNA screening can provide fetal sex information as early as 10 weeks of gestation, with accuracy of ∼99.6% (98.9-100%) [23, 27]. The accuracy of predicting fetal sex by prenatal ultrasound is ∼91% and 99.5%, when performed in the first and the second trimester scan, respectively [28]. These values indicate that in at least 1 out of 200 diploid pregnancies fetal sex prediction might be discordant between cfDNA screening and ultrasound [7]. It has been estimated that the incidence of discordant results between cfDNA and prenatal ultrasound due to differences in sex development is 1 in 1500 to 2000 pregnancies [8], thus only ∼10% of discordant cases are expected to be true positive (XY) or true negative (XX) for Y chromosome cffDNA caused by a DSD.

Aneuploidy, structural rearrangements such as deletions of the Yp encompassing the *SRY* gene (Fig. 1G), and mosaicism involving sex chromosomes are the most common causes of a DSD associated with gonadal dysgenesis. Prenatal cfDNA screening for sex chromosome abnormalities is limited to detection of aneuploidies. Using data from 12 large-scale studies [25, 29-39], we compiled the incidence of prenatal cfDNA-positive samples for sex chromosome aneuploidies, as well as NIPS accuracy characteristics (positive predictive value and FP rate) (Table 3). The overall positive predictive rate of prenatal cfDNA screening for sex chromosome aneuploidies is ∼55%. The rate of DSD-related instances and structural X and Y chromosome rearrangements in pregnancies with a high-risk sex chromosome aneuploidy by prenatal cfDNA screening is anticipated to be higher [26], although the frequency is unknown. The high rate of FP cases, specifically for monosomy X instances, is likely due to causes other than being fetal in origin, but long follow-up studies may provide accurate etiology [39].

**Table 3.** Analytical performance of prenatal cfDNA screening for SCAs

SCA	Estimated incidence in life births	NIPS Incidence*^a^* mean (range)	cfDNA PPV [5]	cfDNA FP rate [5]
45,X	1/10 000 (0.01%)	0.19% (0.09-0.28%)	29.5%	70.5%
47,XXX	5/10 000 (0.05%)	0.09% (0.04-0.18%)	54%	46%
47,XXY	10/10 000 (0.1%)	0.12% (0.06-0.22%)	74%	26%
47,XYY	5/10 000 (0.05%)	0.05% (0.03-0.14%)	74.5%	25.5%

Abbreviations: cfDNA, cell free DNA; FP, false positive; NIPS, noninvasive prenatal screening; PPV, positive predictive value; SCA, sex chromosome aneuploidy.

^*a*^Calculated from the reported noninvasive cfDNA screening results completed on a total of more than 500 000 pregnancies [25, 29-39].

## Inconsistent Prenatal Anatomy Scan and NIPS

A prenatal anatomy scan (a level 2 ultrasound) is usually performed between 18 and 22 weeks of pregnancy, typically after noninvasive cfDNA screening results have become available. The obstetrician benefits from knowing the fetal sex obtained from cfDNA screening. The obstetrician should be familiar with the limitations and accuracy of cfDNA testing and should counsel/refer the pregnant person for additional evaluations. In the absence of pretest counseling, families are typically unaware of the limitations and potential inaccuracies of cfDNA tests [40]. The incidental discovery of discordant fetal sex or atypical genitalia and a possibility of DSD are associated with parental confusion, anxiety, distress, and considerations of pregnancy termination [41, 42]. When faced with inconsistencies between fetal external genital anatomy and NIPS, prompt actions and further evaluation of the fetus are essential to exclude technical errors and other biological causes, and to assess for a DSD. A detailed ultrasound examination might be useful in detecting additional fetal structural abnormalities that may guide future diagnostic strategies and determining chromosomal and monogenic causes of DSDs [43]. Importantly, some anatomic malformations such as bladder or cloacal exstrophy are associated with atypical external genital development; these conditions are independent of the endocrine system.

Concerns related to sample processing, testing laboratory, and ultrasound technical performance should be eliminated first. The most common cause for discordance between prenatal cfDNA screening and ultrasound findings is thought be due to a load of cfDNA from a vanishing twin absorbed into the placenta [9]. Pregnancies achieved by in vitro fertilization with implantation of multiple embryos appear to have a higher rate of discordant results [44].

## Clinical Approach to Situations With Discordant NIPS and Fetal Imaging

### History

When confronted with discordance between NIPS and fetal imaging, the medical history of a pregnant person should be obtained concerning organ transplantation and conditions that may require it. Additional questions include twin siblings, familial incidence of twinning, reproductive and pregnancy history, use of a donor egg, sperm, or an embryo, and preimplantation genetic testing results in cases of in vitro fertilization, the patient's previous genetic testing, including cfDNA finding in prior pregnancies, and exposures to androgens or androgen-modifying drugs (eg, finasteride).

Virilization of XX fetuses may result from gestational hyperandrogenism due to exogenous or endogenous excessive androgen exposure [45]. Virilization symptoms of a pregnant person, including hirsutism and acne worsening, frontal balding, clitoromegaly, and voice deepening, may suggest a luteoma [46], aromatase deficiency, androgen-producing tumors [47], or cytochrome P450 oxidoreductase deficiency. Multiple epidemiological studies have demonstrated that African-American women are more frequently affected by hyperandrogenism in pregnancy than other ethnic groups [48, 49]. The family history may provide insight into genetic disorders and sex chromosome abnormalities [20, 21]. Queries should be made regarding relatives with infertility or abnormal pubertal development and regarding other infants with genital ambiguity or neonatal deaths.

### Prenatal and Postnatal Clinical Management

Recommended actions include repeat cfDNA testing, repeat/review ultrasound findings, and review medical and family history to gather additional information that may explain the discordant results (Tables 1 and 2). Given that pregnant people often choose to learn the biologic sex of their fetus prior to birth, the anomalous anatomy scan and/or NIPS results can be a social emergency. At this point, it is essential to avoid guessing or suggesting possible diagnoses. Parents need to be informed that their child's designated sex of rearing will be determined as rapidly as possible and that they will be involved in this process. However, they need to know that a definitive diagnosis might be established only after birth and might require additional postnatal testing.

If prenatal diagnostic testing is desired, amniocentesis should be performed. Since cfDNA is derived from the placental tissue, prenatal testing using CVS is not useful in determining the chromosomal and genetic status of the fetus. Fetal karyotype and chromosomal microarray analysis (CMA) are recommended, particularly in pregnancies with additional fetal structural anomalies observed by prenatal ultrasound. Additional single gene testing, gene panel, and exome and genome sequencing maybe indicated, depending on the clinical presentation [50]. A prenatal DSD diagnosis provides information about the baby's condition and gives families more time to learn and adjust their expectations [8, 41, 42]. Both karyotype and chromosomal microarray analysis are highly recommended on both biological parents, even if an additional testing for a baby is postponed until birth. In situations where a pregnant person is found to be the carrier of the Y chromosome or its segments, families should be counseled on the recurrent risk of FP or discordant prenatal cfDNA screening results [18, 20, 21].

Comprehensive genetic counseling is essential [42]. Clinicians need to have a dialogue with the parents regarding the possibility of a medical condition, the potential meaning of the atypical anatomic findings, and time constraints especially regarding decision making. Clinicians need to assess the parents’ health literacy and anxiety level to provide appropriate explanations to ensure thorough understanding regarding the test results and their implications. Awareness of the parents’ level of understanding, cultural background, and religious views will enable them to fully participate in decision-making and to provide true “informed consent” [42, 51]. Aspects of these discussions include parents’ expectations about their infant, interpretation of results, and the accuracy of results. Misunderstanding can impact decision-making, potentially resulting in regret regarding decisions.

If technical errors and parental causes are ruled out, consultation with a multidisciplinary DSD team should be promptly arranged. Members of this team include pediatric endocrinologists, pediatric urologists/surgeons, geneticists, neonatologists, radiologists, behavioral health specialists, and pediatric nurse educators. The goal of this team is to assist with the initial decision-making and long-term management of children with DSDs. The postnatal diagnostic evaluation focuses on etiology/diagnosis, appropriate sex designation, and identification of any associated potentially life-threatening conditions such as mineralocorticoid deficiency. Understanding the chromosomal and monogenic factors involved in sex determination and sex differentiation is essential in the evaluation of the fetus and infant with a DSD [43, 52, 53]. The entire clinical picture and the individual patient's needs influence selection of laboratory studies and future personalized clinical management.

## Ethical Considerations

Despite prenatal noninvasive cfDNA analysis being a screening test, with its accuracy impacted by multiple factors as discussed above, families perceive the sex chromosome prediction as being the definitive determination of baby's sex and gender [54]. Genital ambiguity, variation in sex characteristics or discordant genitalia appearance can cause significant parental distress, problems with child bonding, fears regarding child acceptance by family, friends, and community, pressure to make life-changing decisions, and considerations of pregnancy termination due to “quality of life” reasoning. Parental surveys show a high level of unawareness about DSD conditions [8, 42].

Reporting cfDNA results as male or female enforces the binary concept of biological sex, potentially contributing to psychological difficulties in accepting, raising, and supporting a nonbinary child. Discussions should mention that early in development, girls and boys look the same, and that genes and hormones direct the process of genital development. The fetus should be referred to as “your baby” to avoid conveying a sex assignment. Parents should be involved in prenatal and postnatal evaluations. Potential divergence of external and internal genitalia development and gender identity from the sex chromosome status should always be considered in clinical practice and discussed during patient interactions from the beginning of pregnancy.

## Conclusions

Widespread utilization of noninvasive cfDNA screening for all pregnant individuals is a technological achievement with an enormous positive impact on prenatal diagnosis. In addition to screening for common autosomal trisomies (chromosomes 13, 18, and 21), NIPS provides an opportunity to identify possible fetal and parental sex chromosome variations, suspect other health-relevant conditions, and predict fetal sex. Despite its complexity, variability in performance of various cfDNA methodologies, and accuracy in detection of different chromosomal conditions, cfDNA testing advances our knowledge in the field of reproductive medicine and human biology. Awareness among clinicians regarding a possible discordance between fetal sex predicted by prenatal cfDNA screening or prenatal ultrasound and actual phenotype and sex chromosomes at birth is essential. Such knowledge will help update the definitions of sex [55] to break down stereotypes, create an inclusive and supportive society, and establish ethical and competent healthcare.

### Highlights

- Neither noninvasive cfDNA screening nor prenatal ultrasound is 100% accurate in fetal sex prediction.
- Most discrepant sex chromosome cfDNA results are not due to fetal causes; however, up to ∼30% of instances require evaluation for DSDs.
- Substantial knowledge gaps regarding accuracy and implications of fetal sex predictions exist among healthcare professionals and patients.
- Educational resources and test performance characteristics for fetal sex prediction should be available to patients prior to cfDNA screening to minimize the negative psychosocial impact upon discovery of discrepant results.

## Disclosures

Dr. Selma Witchel is an Editorial Board Member for Journal of the Endocrine Society and played no role in the Journal's evaluation of the manuscript.

## Data Availability

Data sharing is not applicable to this article as no datasets were generated or analyzed during the current study.

## Abbreviations

cfDNA: cell free DNA
cffDNA: fetal cell free DNA
CMA: chromosomal microarray analysis
CVS: chorionic villus sampling
DSD: difference of sex development
FF: fetal fraction
FN: false negative
FP: false positive
MPSS: massively parallel shotgun sequencing
NIPS: noninvasive prenatal screening
NIPT: noninvasive prenatal testing
SNP: single nucleotide polymorphism
